# A Humanized Anti-GPC3 Antibody for Immuno-Positron Emission Tomography Imaging of Orthotopic Mouse Model of Patient-Derived Hepatocellular Carcinoma Xenografts

**DOI:** 10.3390/cancers13163977

**Published:** 2021-08-06

**Authors:** Arutselvan Natarajan, Hui Zhang, Wei Ye, Lakshmi Huttad, Mingdian Tan, Mei-Sze Chua, Sanjiv S. Gambhir, Samuel K. So

**Affiliations:** 1 Molecular Imaging Program at Stanford (MIPS), Department of Radiology and Bio-X Program, Stanford University, Stanford, CA 94305, USA; sgambhir@stanford.edu; 2 Asian Liver Center, Department of Surgery, School of Medicine, Stanford, CA 94305, USA; huizhang0511@gmail.com (H.Z.); yeweistanford@gmail.com (W.Y.); lhuttad@stanford.edu (L.H.); mdtan@stanford.edu (M.T.); samso@stanford.edu (S.K.S.)

**Keywords:** immunoPET imaging, humanized antibody, glypican-3, hepatocellular carcinoma

## Abstract

**Simple Summary:**

Liver cancer, the majority of which is hepatocellular carcinoma, is a typically fatal adult liver malignancy. It is hard to detect in the early stages of the cancer, and therefore patients are often diagnosed at the advanced stages, when treatment options become more limited and survival outcomes are poor. To improve early detection, and therefore treatment and prognosis of liver cancer patients, we have developed an imaging probe for positron emission tomography, targeting a protein, glypican-3, which is specifically expressed at high levels in liver cancer cells. Our probe consists of the ^89^Zr radioisotope conjugated to a humanized monoclonal antibody against glypican-3, and it demonstrates specific ability to detect patient-derived liver cancer xenografts in a mouse model. With a high tumor to normal liver contrast, we believe this imaging probe can provide a useful tool in the early diagnosis and timely medical intervention for liver cancer patients.

**Abstract:**

Glypican-3 (GPC3) is an attractive diagnostic marker for hepatocellular carcinoma (HCC). We previously reported the potential of an ^89^Zr-labeled murine anti-GPC3 antibody (clone 1G12) for immunoPET imaging of HCC in orthotopic patient-derived xenograft (PDX) mouse models. We now humanized the murine antibody by complementarity determining region (CDR) grafting, to allow its clinical translation for human use. The engineered humanized anti-GPC3 antibody, clone H3K3, retained comparable binding affinity and specificity to human GPC3. H3K3 was conjugated with desferrioxamine (Df) and radiolabeled with ^89^Zr to produce the PET/CT tracer ^89^Zr-Df-H3K3. When injected into GPC3-expressing orthotopic HCC PDX in NOD SCID Gamma (NSG) mice, ^89^Zr-Df-H3K3 showed specific high uptake into the orthotopic PDX and minimal, non-specific uptake into the non-tumor bearing liver. Specificity was demonstrated by significantly higher uptake of ^89^Zr-Df-H3K3 into the non-blocked PDX mice, compared with the blocked PDX mice (which received prior injection of 100 mg of unlabeled H3K3). Region of interest (ROI) analysis showed that the PDX/non-tumor liver ratio was highest (mean ± SD: 3.4 ± 0.31) at 168 h post injection; this ratio was consistent with biodistribution studies at the same time point. Thus, our humanized anti-GPC3 antibody, H3K3, shows encouraging potential for use as an immunoPET tracer for diagnostic imaging of HCC patients.

## 1. Introduction

Hepatocellular carcinoma (HCC) is the sixth most common cancer globally and the fourth leading cause of cancer-related death: In 2018, there were an estimated 841,000 new HCC patients diagnosed and an almost equal number of deaths (782,000) globally, leading to a mortality rate of 0.93 [1]. This high mortality rate is partially attributed to the asymptomatic nature of the disease, with many patients not presenting with symptoms until the late stages, and is compounded by the lack of effective treatment options at the late stages. Detection of HCC at an early stage when it is amenable to treatment by surgical resection or liver transplantation is crucial to the improvement of the survival rates of HCC patients [2].

Clinically, HCC diagnosis is based on biomarker serology and radiology. Unfortunately, these tests (alone or in combination) lack high specificity and sensitivity for identifying early HCC. Alpha-fetoprotein (AFP), a serologic biomarker used for decades to screen and diagnose HCC, is currently not recommended by American and European guidelines [2,3] due to the inadequate sensitivity (61%) and specificity (71%) when combined with ultrasound. Other recent emerging biomarkers, such as lectin-binding AFP and des-gamma carboxyprothrombin (DCP), have poor sensitivity (<40%) and specificity (<92%), which make them unreliable [4,5,6]. Current diagnosis of HCC heavily relies on radiology methods, such as ultrasound, computerized tomography (CT) scan, and magnetic resonance imaging (MRI). However, these modalities have limited size resolution and difficulties in differentiating malignant HCC from benign liver lesions. Ultrasound can detect only 60% of early HCC in high-risk cirrhosis patients [4,7]. Both CT and MRI provide 100% sensitivity for nodular HCC larger than 2 cm and around 40% sensitivity for 1–2 cm nodules but have poor sensitivity for lesions smaller than 1 cm (10–33% for CT and 29–43% for MRI) [6]. Since early detection of HCC is critical for timely treatment, improved prognosis, and survival, it is imperative to develop methods for HCC diagnosis with enhanced sensitivity and specificity, which may also be valuable for the monitoring of treatment response and tumor recurrence.

GPC3 is a cell surface heparin sulfate proteoglycan consisting of a core protein anchored to the cytoplasmic membrane via a glycosyl phosphatidylinositol linkage. Recent research has established the role of GPC3 as a promising biomarker for HCC since it is over-expressed in greater than 50% of HCC patients [8,9,10]. GPC3 is reported to be more specific and reliable than other blood-based biomarkers (including AFP) in the detection of HCC [11,12,13]. Its membrane location makes it readily accessible for antibody-based diagnostic and therapeutic approaches for HCC. An important characteristic of GPC3 is its preferential over-expression in malignant HCC cells, compared with other pre-neoplastic or benign liver lesions or in cirrhosis [11]. By allowing earlier confirmatory diagnosis of HCC, GPC3-based molecular imaging modalities have the potential to allow timely medical intervention to increase the overall survival rate of HCC patients. Lastly, given the functional roles of GPC3 in regulating various signaling pathways that are hyper activated in HCC [12], the ability to accurately detect GPC3-positive HCC tumors using immunoPET offers a valuable tool for monitoring the response of HCC treated with targeted therapy against GPC3 [13].

We previously provided proof-of-concept that PET imaging of HCC using ^89^Zr-radiolabeled murine anti-GPC3 antibody (clone 1G12) could successfully delineate orthotopic patient-derived HCC xenografts from normal liver [14]. With its long half-life, ^89^Zr (78.4 h) was able to overcome the typical challenges of high liver background, resulting in clinically acceptable tumor-to-liver ratios (>2) and excellent contrast of the tumor from the adjacent non-tumor liver. However, the clinical utility of this antibody is limited by its murine origin. Here, we report the successful use of an immunoPET probe based on ^89^Zr-radiolabeled, humanized anti-GPC3 monoclonal antibody (clone H3K3, obtained through complementarity determining region (CDR) grafting of the murine 1G12 clone) to accurately identify GPC3-positive HCC cells in vitro and in vivo. The encouraging in vivo PET imaging performance of this humanized probe makes it highly promising for clinical translation.

## 2. Results

### 2.1. Humanized Anti-GPC3 Antibody H3K3 Retains Specificity and Binding Affinity and to GPC3

**Figure 1:**
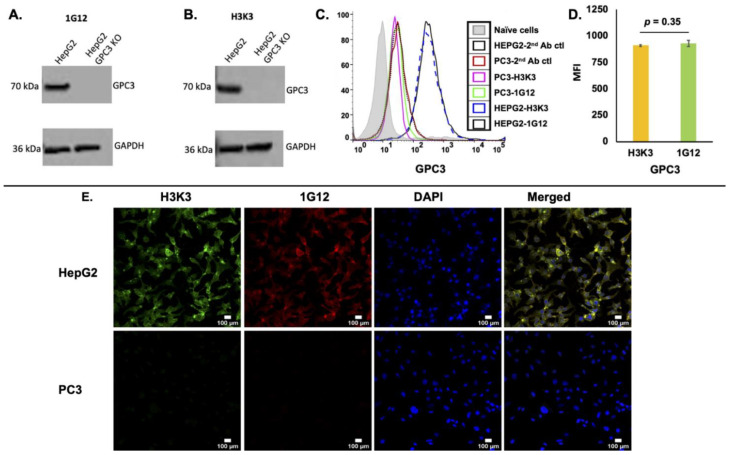
Clone H3K3 shows greatest binding specificity to GPC3 in HepG2 cells. Western blot of (**A**) mouse clone 1G12 and (**B**) humanized clone H3K3 staining of GPC3 expression in HepG2 parental cells (GPC3-positive) and GPC3-knockout counterparts (HepG2 GPC3KO; GPC3 negative). GAPDH was used as the internal control. (**C**) Analytical flow cytometry of surface GPC3 expression on HepG2 with 1G12 (black line) or H3K3 (blue dotted line). Naive cells control (grey filled area, without adding primary and secondary antibodies); and secondary antibody alone with HepG2 and PC3 (black dotted line, and red line, respectively) were used as the controls. (**D**) Measure of mean florescence intensity (MFI) of GPC3 expression in HepG2 cells measured by H3K3 and 1G12 antibodies. (**E**) Immunofluorescence staining with H3K3 (in green), 1G12 (in red), and DAPI (in blue) in HepG2 (GPC3-positive) and PC3 (GPC3-negative) cells, respectively. Co-localization of H3K3 and 1G12 staining was visualized as yellow staining in the merged panels. Scale bars shown for 100 μm.

A humanized anti-human GPC3 antibody (clone H3K3) was generated by CDR grafting based on its parental mouse 1G12 clone (this work was done by Creative Biolabs, Shirley, NY, USA) (Figure S1). H3K3 was tested for specific binding to GPC3 in the HCC cell line HepG2, which expresses high levels of GPC3. H3K3 showed similar specificity as its original mouse 1G12 clone in its ability to detect GPC3 in HepG2 (WT) cell lysate but not in HepG2 cells with GPC3 knockout (GPC3KO) (Figure 1A,B; Figure S2). When analytical flow cytometry was performed to compare binding specificity of H3K3 with its original mouse 1G12 clone, H3K3 demonstrated a similar extent of binding as 1G12 to GPC3 expressed on the surface of HepG2 (Figure 1C,D). To further confirm the binding specificity of H3K3, immunofluorescence staining was performed on HepG2 and PC3 cells (a prostate cancer cell line without GPC3 expression) [14]. Confocal microscopy showed that H3K3 staining was co-localized with 1G12 staining in HepG2 cells, while showing no signals in PC3 cells (Figure 1E). To determine that H3K3 retains binding affinity to GPC3 (compared with 1G12), we performed in vitro binding assay in the GPC3 positive HepG2 cells. Both H3K3 and 1G12 were biotinylated and stained with streptavidin-APC fluorophore, and their respective Kd (nM ± SD, *n* = 2) values were 3.89 ± 0.23 and nM 3.50 ± 0.35 nM (*R^2^* = 0.99) (Figure S3). Both antibodies did not bind significantly to the GPC3 negative PC3 cells.

### 2.2. Synthesis and Quality Assurance of Pre-Cursor (Df-H3K3) and PET Tracer (^89^Zr-Df-H3K3)

As a first step to synthesizing the PET tracer ^89^Zr-Df-H3K3, we first covalently coupled Df-Bz-NCS (Df) to the lysine groups of H3K3 at pH 8.0, using a five-molar excess of Df to H3K3. We were able to reproducibly yield a ratio of 1.8 ± 0.2 (mean ± SD, *n* = 3) Df-chelates per H3K3 antibody, as confirmed by MALDI-TOF. Radiolabeling of Df-H3K3 with ^89^Zr resulted in radiochemical yield of >70% of ^89^Zr-Df-H3K3. Purification of ^89^Zr-Df-H3K3 by HPLC resulted in purity >95% of monomeric antibody (mean ± SD: 96.9 ± 2.2%), with specific activity of 33 ± 9 MBq/nmol. Table 1 shows the quality assurance characteristics of the ^89^Zr-Df-H3K3 tracer (*n* = 3). The ^89^Zr-Df-H3K3 tracer demonstrated 69.6 ± 3.2% (mean ± SD) immunoreactivity towards HCC PDX622 cells that express GPC3 (Figure S4). These data confirmed the high quality and specificity of ^89^Zr-Df-H3K3, which makes it a suitable immunoPET tracer for targeting human GPC3 expressed in orthotopic HCC PDX mouse models.

### 2.3. ImmunoPET Imaging Using ^89^Zr-Df-H3K3 Detects Orthotopic HCC PDX with Minimal Normal Liver Background

**Figure 2:**
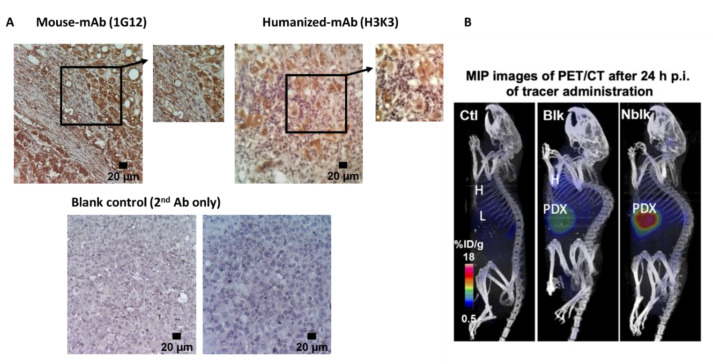
PET/CT imaging of GPC3-expressing orthotopic HCC PDX in mice using ^89^Zr-Df-H3K3. (**A**) Immunohistochemistry detection of GPC3 in orthotopic HCC PDX tissues harvested from mice, using original mouse 1G12 clone (left image) and the humanized H3K3 clone (right image). Bottom panel shows negative controls using secondary (2°) antibody only for staining. Magnification of 20× for images (scale bars of 20 μm); 100× for enlarged insets. (**B**) Representative PET/CT images from three groups of mice (*n* = 4): non-tumor control (left), blocking PDX-mice (middle), and non-blocking PDX-mice (right) at 24 p.i. of ^89^Zr-Df-H3K3 tracer (100 µCi in 200 µL).

Using IHC, we confirmed that H3K3 antibody was able to detect GPC3 in HCC PDX tissues equally well as the parental mouse 1G12 antibody [15] (Figure 2A, right and left panels, respectively; with secondary antibody blank controls shown in the bottom panels). 

ImmunoPET scans were performed at various time points (4–168 h) post injection (p.i.) of 3.7 ± 0.4 MBq or 100 µCi of ^89^Zr-Df-H3K3. PET images were co-registered with CT to visualize the mouse organs clearly. Representative maximum intensity projection (MIP, sagittal) PET/CT images of NSG mice (Ctl, PDX-blk, and PDX-nblk) at 24 h p.i. showed accumulation of ^89^Zr-Df-H3K3 in the orthotopic HCC PDX in the PDX-nblk mice (Figure 2B, right panel). Competitive blocking group received with 15-fold excess of cold H3K3, that reduced the uptake of radioactivity by over 50% in the PDX-blk mice, demonstrating specificity of the tracer (Figure 2B, middle panel). Minimal background radioactivity was observed in the liver of the non-tumor bearing control mice when compared with the non-tumor liver of the PDX mice (Figure 2B, left panel).

**Figure 3:**
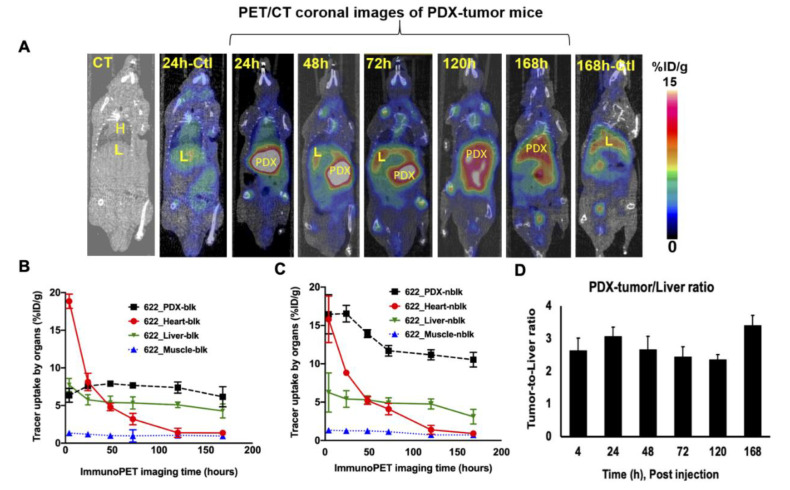
PET/CT coronal images of ^89^Zr-Df-H3K3 in PDX-NSG and control mice. (**A**) PET/CT coronal images from PDX-NSG at various time points (24, 48, 120, 72, and 168 h p.i.) after administration of ^89^Zr-Df-H3K3. PET/CT or CT images of control group are also shown (at 24 h and 168 h p.i.) for comparison. Data were computed as mean ± SD %ID/g (*n* = 4) from ROI of non-tumor bearing liver from control group. (**B**,**C**) Time-activity curves of ^89^Zr-Df-H3K3 uptake into the PDX, heart, liver and muscle in the (**B**) blocking and (**C**) non-blocking mice groups were computed as ROI from the PET images. (**D**) PDX-to-liver ratio of non-blocking mice at various time points were computed from ROI data. Tracer uptake are decay corrected and presented as mean ± SD %ID/g.

Representative PET/CT coronal images of PDX-NSG mouse group at various time points (24, 48, 72, 120, and 168 h p.i.) are shown in Figure 3A, and non-PDX control mouse images are also displayed (24 and 168 h p.i.). Uptake of the tracer into the PDX, heart, liver, and muscle derived from ROI of PET images at various time points (24, 48, 72, 120 and 168 h p.i.) are shown in Figure 3B,C for blocking and non-blocking groups, respectively. While tracer uptake into heart, liver, and muscle was similar in both blocking and non-blocking groups, uptake into the PDX was at least 1.5-fold higher in the non-blocking group throughout the entire period, and remained elevated in the PDX even at 168 h. Tracer uptake into the PDX of the blocking group remained low (between 6 %ID/g and 8 %ID/g) throughout the entire period. PDX-to-liver ratios at various time points also suggest at least 2.0-fold higher uptake in the PDX compared with the non-tumor liver at all time points, with the highest ratio (3.4 ± 0.31) at 168 h (Figure 3D). At 24 h p.i., tracer uptake into the PDX was 16.5 ± 1.1 %ID/g in the non-blocked group, compared with 7.6 ± 0.3 %ID/g in the blocked group (*p* = 0.003); tracer uptake into the corresponding non-tumor livers was 5.4% ± 0.2 %ID/g in the non-blocked group and 5.7% ± 0.7 %ID/g in the blocked group. The tracer uptake into the normal liver of the non-tumor control group was highly consistent at 5.4 ± 0.6 %ID/g in the non-tumor bearing liver in the control group (*p* = 0.001). Thus, tracer uptake was 3-fold higher in the PDX compared with adjacent non-tumor liver, as well as normal liver in control mice, providing evidence of specific targeting of tracer to PDX in vivo. PET signals arising from the non-tumor bearing liver of control mice could be non-specific due to slow hepatic clearance of the immunoPET tracer.

**Figure 4:**
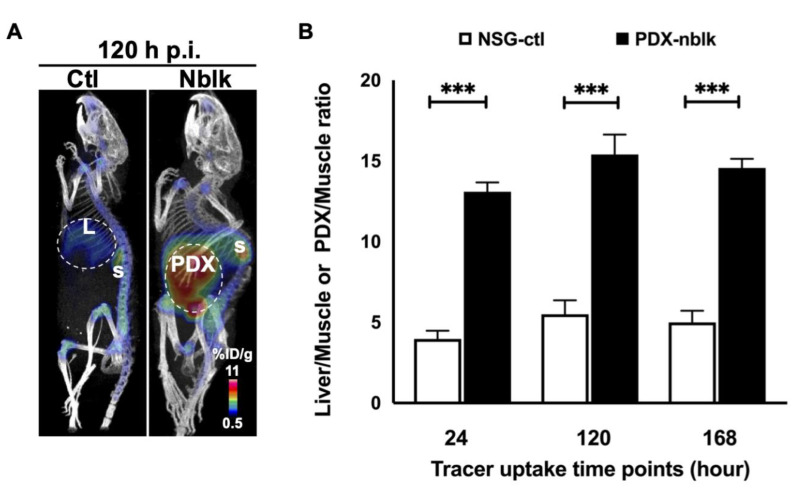
PET/CT images at 120 h p.i. of ^89^Zr-Df-H3K3 and Ctl liver-to-muscle or PDX-to-muscle ratios. (**A**) PET/CT images of orthotopic HCC PDX at 120 h p.i. showed marked accumulation of ^89^Zr-Df-H3K3 in the PDX of the non-blocking group compared with the non-tumor bearing liver (L) of the control group. S = Spleen (**B**) Ctl liver-to-muscle or PDX-to-muscle ratios at various time points (24, 120, and 168 h p.i.) of ^89^Zr-Df-H3K3. Data were computed as mean ± SD %ID/g (*n* = 4) from ROI of non-tumor bearing liver from control group, and of the PDX tissues in the non-blocking PDX group. *** *p* = 0.001.

At 120 h p.i. of the tracer, representative coronal immunoPET images showed significantly greater retention of radioactivity in the PDX of the non-blocked mice than in the non-tumor bearing liver of the control mice (Figure 4A). At 24 h, 120 h, and 168 h p.i., the PDX-to-muscle ratios in the non-blocked group were all about 3-fold higher (*p* < 0.0005) than the non-tumor bearing liver-to-muscle ratios in the control group (Figure 4B). For example, at 168 h p.i., the PDX-to-muscle ratio in the non-blocked group (14.6 ± 0.6) was 2.9-fold higher than that in the control group (5.0 ± 0.7). The spleen uptake of PDX-NSG-nblk was between 5 %ID/g and 7 %ID/g at 4 h and 168 h, respectively.

### 2.4. Biodistribution of ^89^Zr-Df-H3K3 into PDX and Various Mouse Organs

**Figure 5:**
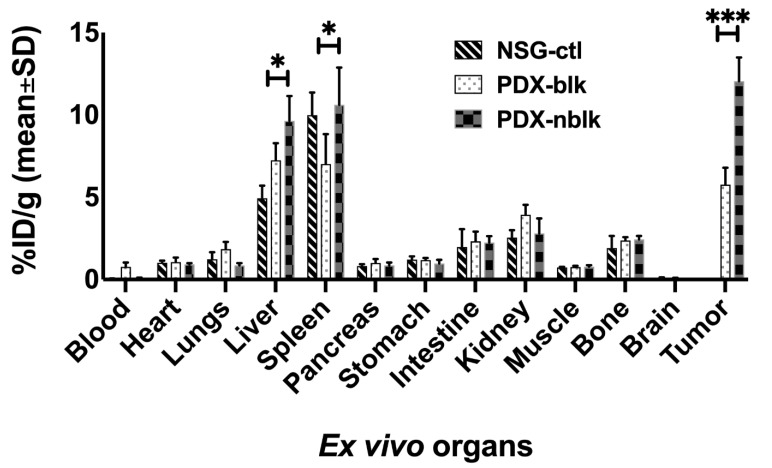
Biodistribution measurements of ^89^Zr-Df-H3K3 tracer uptake. Major organs were harvested at 168 h p.i. of ^89^Zr-Df-H3K3, and ex vivo tracer uptake into each organ was measured using the gamma counter: NSG liver (Ctl); PDX mice liver-tumor blocked with pre-dose (Blk), and non-blocking liver-tumor (Nblk). Data are presented as mean ± SD %ID/g in four mice per group (NSG-ctl with non-tumor bearing liver; PDX-NSG-blk for blocking group; and PDX-NSG-nblk for non-blocking group). * *p* = 0.05, *** *p* = 0.001.

At the end of the immunoPET/CT scans (at 168 h p.i.), mice were euthanized and the PDX and various organs were harvested to determine biodistribution of the ^89^Zr-Df-H3K3 tracer (Figure 5, Tables S1 and S2). Tracer uptake in the PDX harvested from the non-blocked group of mice (PDX-NSG-nblk; 12.1 ± 1.4% ID/g) was over 2-fold higher than that in the PDX from blocked mice (PDX-NSG-blk; 5.7± 1.0% ID/g) and in the non-tumor bearing liver of the control mice (4.9 ± 0.7% ID/g). Consistent with ROI quantification of PET/CT images (Figure 4B), biodistribution analysis indicated that the PDX-to-muscle ratio in the non-blocked mice was 15.46 ± 1.16% ID/g, which is 2.3-fold higher than the non-tumor bearing liver-to-muscle ratio in the control mice was 6.73 ± 0.75 %ID/g (*p* = 0.0001). Our data suggest that tracer uptake was <3% in most of the studied organs, except in the kidney, liver, and spleen. Tracer uptake in the liver and spleen may result from the antibody being cleared from these major clearance organs; uptake in the kidney and intestine may be due to expression of GPC3 in the normal kidney and intestine [16].

## 3. Discussion

This study extends our earlier report on using a radiolabeled (^89^Zr) murine anti-GPC3 antibody (clone 1G12) for successful immunoPET imaging of orthotopic HCC PDX [14]. We have demonstrated that the humanized 1G12 (clone H3K3) retains comparable binding affinity and specificity to native GPC3 in HCC cells and that it can similarly be successfully radioconjugated with ^89^Zr for immunoPET imaging of the same orthotopic HCC PDX model. We continue to use ^89^Zr as the preferred radioisotope, as its long half-life (3.3 days; 78.4 h) matches the clearance profile of a full-length antibody (which has a relatively slow pharmacokinetics, with half-life of about 5–7 days), thereby achieving clinically favorable tumor-to-liver ratios.

The humanized H3K3 retains the same CDR as 1G12 (Figure S1); additionally, the FR (framework region) is highly conserved: the consensus homology between H3K3 and 1G12 in the VH region is 94.8% (with 87.8% identical sequences), and in the VK region, it is 97.3% (with 92.0% identical sequences). As such, we had expected H3K3 to possess equivalent specificity and binding affinity to human GPC3 (which has 96.2% consensus positions and 81.3% identity positions as mouse GPC3). Indeed, our in vitro binding results indicate that humanization of 1G12 did not negatively alter the binding affinity of H3K3 to GPC3. As the CDR regions between H3K3 and parental1G12 are the same, it is likely that the humanized FR regions contributed to the enhanced affinity, perhaps by promoting a more favorable conformation of the humanized IgG, which may enhance binding to GPC3. H3K3 performed equally well as 1G12 in detecting native GPC3 in an HCC cell line, as confirmed by in vitro cell binding assay, Western blotting, FACS, and immunofluorescence staining. Furthermore, H3K3 was readily amenable to bioconjugation and radiolabeling, generating a high purity immunoPET tracer, ^89^Zr-Df-H3K3, that retains 69% immunoreactivity against GPC3-expressing HCC PDX cells.

As the liver is largely responsible for the clearance of exogenous molecules, a typical high liver background is observed with most immunoPET tracers, which is a major hurdle in the clinical translation of immunoPET probes for HCC diagnosis. The ^89^Zr-Df-H3K3 tracer demonstrated specific binding to the GPC3-expressing PDX, as determined through competitive blocking with an excess of cold H3K3, which reduced the uptake of radioactivity by over 50% in the PDX-blk mice. The tracer also accumulated readily within the PDX, as early as 24 h p.i., showing 3-fold higher accumulation in PDX compared with both adjacent non-tumor livers and in normal livers of non-tumor bearing control mice. Importantly, the tumor-to-liver ratio was >3.0 at 168 h p.i., consistent with our earlier study using the parental 1G12 antibody [14]. Ex vivo biodistribution studies (at 168 h p.i) consistently showed a 3-fold higher tracer uptake in the PDX of non-blocked mice, compared with the non-tumor bearing liver of control mice (Figure 5). There was minimal tracer uptake into major organs such as the heart, brain, lungs, muscle, and bone. Bone uptake, which is <5% ID/g, could be observed in some joints, and this may be caused by accumulation of free ^89^Zr in the bone marrow, due to instability of chelation with DFO [17,18]. This phenomenon was also observed in immuno-PET imaging of other types of cancers using DFO as the chelator for ^89^Zr [19]. The observed liver uptake may have resulted from remnant PDX cells that were not completely dissected from the rest of the liver. Collectively, these in vivo observations suggest that the ^89^Zr-Df-H3K3 tracer is a potentially promising immunoPET probe that can overcome the challenge of high non-specific liver background.

Although other groups have developed various diagnostic agents to target GPC3, such as other mouse anti-human anti-GPC3 antibodies [20] and peptide- or aptamers-based tracers [21,22,23], all have suffered limitations to clinical translations. To the best of our knowledge, there has been no clinical study yet reported to use fully humanized anti-GPC3 antibody or a humanized anti-GPC3 antibody. Compared with intact antibodies, aptamers- and peptide-based anti-GPC3 probes demonstrated high specificity and binding affinity; however, they were evaluated using fluorescence dyes, which limits their clinical use due to poor penetration of light into and from deep tissues [21,22,23]. Other peptide-based probes labeled with ^18^F showed high uptake with high tumor/muscle ratio but also correspondingly high uptake into the non-tumor liver, resulting in poor tumor-to-liver contrast (0.93 ± 0.16), which is of limited use for detecting liver tumors [23]. ImmunoPET tracers using intact anti-GPC3 antibodies can achieve high specific uptake at the tumor sites. Even though achieving high tumor-to-liver contrast can be challenging [14], we and others have shown that this can be overcome by using longer half-life radioisotopes to allow imaging several days after tracer injection, when the unbound tracer has been cleared [20]. Hanaoka et al. labeled a human heavy chain GPC3 antibody (HN3) with ^125^I [24] and demonstrated rapid tumor internalization of this tracer, together with rapid blood clearance and improved homogeneity within the tumor compared with the full IgG antibody. However, it showed lower immunoreactivity (since the critical antigen-binding tyrosine residues are subject to iodination) and high renal accumulation, which was not observed in our study. Based on our data, we believe that an immunoPET tracer based on the ^89^Zr radioisotope can achieve clinically desirable tumor-to-liver and tumor-to-muscle ratios.

We have successfully demonstrated that a humanized anti-GPC3 antibody can be developed as a sensitive immunoPET tracer for HCC detection. Further optimization will be needed to enhance the immunoreactivity of ^89^Zr-Df-H3K3 above the current 69%, as well as to determine its dosimetry, shelf life, and toxicity so that we can determine a safe and effective dose for in-human use [25]. Additionally, we will further confirm that there is no or minimal cross reactivity between GPC3 and other glypican family members to ensure that there is no non-specific background or toxicity. We anticipate such cross reactivity to be low, given that the amino acid identify was reported to be about 25% among the six glypican members (GPC1 to GPC6) [26]. The successful clinical translation of this tracer offers a potentially valuable tool for the early diagnosis of HCC since GPC3 is reported to be expressed in HCC cells in the early stages of malignant transformation [27]. The ability of the tracer to detect small HCC lesions from surrounding non-tumor liver will be a focus of future clinical studies. Additionally, since GPC3 is over-expressed in over 50% of HCC patients (regardless of viral etiology), it is a potentially useful diagnostic imaging marker that can complement the traditionally used AFP blood biomarker. Clinically, we envision that ^89^Zr-Df-H3K3 can be used to identify GPC3-positive HCC patients, who can then be offered GPC3-targeted therapy (such as radioimmunotherapy). Subsequently, monitoring of treatment response and tumor recurrence would be performed using ^89^Zr-Df-H3K3. Additionally, our finding that a sufficiently high tumor-to-liver contrast could be achieved 24 h p.i. suggests that PET acquisition can be performed on HCC patients at this earlier time point in clinical practice. It also suggests the possibility of using a shorter half-life radioisotope, ^64^Cu, that has been FDA approved for PET imaging in human cancers [28]. To this end, our follow-up study will comprehensively evaluate the in vivo performance of an ^89^Zr vs. ^64^Cu-labeled H3K3 immunoPET probe, simultaneously comparing their tumor uptake, tumor-to-liver contrast, and biodistribution at 24 h p.i. These data will guide us in the selection of the radioisotope to be used in clinical translation of the H3K3 immunoPET probe.

Last but not least, over-expression of GPC3 in HCC patients has been associated with poorer overall survival and disease-free survival [29]; thus, by offering sensitive and non-invasive detection of early-stage HCC (that is GPC3 positive), our GPC3-targeted imaging can potentially increase patient survival through timely intervention and through monitoring of treatment response and tumor progression. Furthermore, the humanized H3K3 antibody can also be developed for radioimmunotherapy, such as for selective delivery of therapeutic radioisotopes (e.g., ^90^Y or ^177^Lu). In this regard, the current probe ^89^Zr-Df-H3K3 can be used to estimate retention in the tumor, as well as to provide insights on side effects in at-risk organs by a companion dosimetric approach. Desirable characteristics of a radiopharmaceutical, such as rapid tumor internalization and rapid blood clearance, will be assessed to determine the ideal agent to be used for GPC3-targeted radioimmunotherapy of HCC.

## 4. Materials and Methods

### 4.1. Cell Culture

HepG2 and PC3 cell lines were purchased from American Type Culture Collection (ATCC, Manassas, VA, USA). HepG2 GPC3 knockout (KO) cells were established as previously described [30]. All cell lines were cultured in Eagle’s Minimum Essential Medium (ATCC, Manassas, VA, USA) supplemented with 10% fetal bovine serum, 100 μg mL^−1^ penicillin, and 100 μg mL^−1^ streptomycin) (all supplements were obtained from Life Technologies (Carlsbad, CA, USA). Cells were maintained in a humidified atmosphere of 5% CO_2_ at 37 °C.

### 4.2. Humanization of Mouse Anti-GPC3 (Clone 1G12) by CDR Grafting and Purification of Humanized H3K3 Clone

This work was performed by Creative Biolabs (Shirley, NY, USA) by inserting the CDR of mouse origin anti-GPC3 antibody (clone 1G12) into the human antibody scaffold (Figure S1). Briefly, sequencing of genomic DNA was first performed on the cell lysates from hybridoma cells expressing clone 1G12, provided by BioMosaics Inc. (Burlington, VT, USA). Murine-sequence derived CDRs were identified and engrafted into the expression cassette of humanized IgG (the heavy chain cassette was pCMV-HindIII-Kozak-leader-VH-CH123-BamHI-BGH polyA; the light chain cassette was pCMV-HindIII-Kozak-leader-VL-CK-BamHI-BGH polyA). Humanized 1G12 (clone H3K3) was obtained through CDR grafting and proper back mutation (a total of seven backmutations), in which the CDR regions and approximately 90% of framework residues from paternal 1G12 were retained. Constructs were transfected into HEK293T cells and ELISA was performed to confirm binding activity with recombinant human GPC3. Prior to radiochemistry, H3K3 was purified using Protein G column (Thermo Fisher, Rockford, IL, USA) with elution buffer (0.1 M glycine, pH 2.0). Microcon 30 (Millipore, Ireland, UK) was then used to concentrate H3K3 by spinning down several times at 300 g, 20 min each spin, at 4 °C. The purity of H3K3 was confirmed by NuPAGE 4–12% Bis-Tris protein gel (ThermoFisher Scientific, Carlsbad, CA, USA), followed by Coomassie Blue (Bio-Rad, Hercules, CA, USA) staining and Superdex 200 10/300 GL (GE Healthcare Life Sciences, Uppsala, Sweden) column with ÄKTA pure protein purification system (GE Healthcare Life Sciences, Uppsala, Sweden). The yield was >80%.

### 4.3. Synthesis of ^89^Zr-Df-H3K3 ImmunoPET Tracer

The purified humanized anti-GPC3 antibody H3K3 was first buffer exchanged with 1 M HEPES/0.1 M Na_2_CO_3_ (pH 8.5 ± 0.5) and concentrated to ~3 mg/mL using a Vivaspin 30 kDa centrifugal filter (Thermo Fisher Scientific, Waltham, MA, USA; Catalogue #VS2021). H3K3 was then conjugated with *p*-isothiocyanatobenzyl-desferrioxamine (Df-Bz-NCS) (Macrocyclics, Dallas, TX, USA) by mixing an aliquot (~1.7 mL) of 33.3 nmol/mL of H3K3 (20 × M in 1 M HEPES buffer solution, pH 8.5–9.0) with 5 molar excess of the Df-Bz-NCS (10 mM dissolved in DMSO; 7.5 mg/mL, ~20 μL) at 37 °C for 60 min. High-performance liquid chromatography (HPLC) performed on HPLC-Ultimate (Thermo Fisher Scientific, Waltham, MA, USA) was used to remove excess unconjugated Df-Bz-NCS using 0.1 M ammonium acetate buffer (pH 6.5) as the mobile phase, eluted at 1 mL/min. The immunoconjugate was concentrated to ~2 mg/mL using a Vivaspin, 30 kDa cutoff centrifugal filter and stored in 200 μL aliquots in 0.1 M ammonium acetate buffer (pH 5.5) at −4 °C. The number of chelators (c) coupled per antibody (a), i.e., c/a was estimated with matrix-assisted laser desorption/ionization time-of-flight mass spectrometry (MALDI-TOF-MS) in comparison with unmodified H3K3 and Df-H3K3.

Finally, ^89^Zr isotope (220–230 MBq; 500 μL) was mixed with 100–200 × L of 0.1 M oxalic acid, followed by 1 M Na_2_CO_3_ (80 ± 20 μL), and kept at room temperature for 3 min. HEPES buffer (0.5 M, pH 7 ± 0.5; 300 ± 20 μL) and Df-H3K3 (2 mg; 1 mL) were then added to the ^89^Zr solution and the pH was readjusted to 7 ± 0.5 using 0.5 M HEPES. The reaction mixture was then incubated at 37 °C for 60 min. The purity of the radiotracers was tested by thin layer chromatography as well as by SEC-2000 radio-HPLC.

### 4.4. Establishing Orthotopic Xenografts from HCC Patient Tumors

HCC tissues were collected from HCC patients who had undergone liver resection as part of their treatment. This study was approved by the Institutional Review Board at Stanford University for the use of human subjects in medical research, and informed consent was obtained from each patient prior to liver resection. All experimental protocols involving animals were approved by the Institutional Animal Care and Use Committee (the Stanford Administrative Panel on Laboratory Animals Care (APLAC)). All animal studies were carried out in compliance with the approved protocols and were in compliance with the ARRIVE guidelines.

Orthotopic xenografts from HCC patient tumors were established and monitored with bioluminescence imaging as previously described [14,31]. As a first step, HCC patient tumor tissue (generating PDX622) was digested to form single cell suspension and transduced with firefly luciferase [31]. PDX622 cells expressing luciferase were resuspended in 100 μL of Dulbecco’s Phosphate Buffered Saline (DPBS) (Invitrogen Life Technologies, Carlsbad, CA, USA) and mixed with 100 μL Corning Matrigel Membrane Matrix (354234, Corning, Bedford, MA, USA) for subcutaneous injection near the left shoulder of 6- to 8-week-old male NOD Cg-*Prkdc^scid^ Il2rg^tm1Wjl^*/SzJ mice (NSG mice, Charles River Laboratories Inc., Cambridge, MA, USA). Once the subcutaneous PDX was established, xenografts were harvested and cut into ~2 mm^3^ pieces for surgical implantation onto the left lobe of the liver of another group of 6- to 8-week-old male NSG mice (*n* = 4). Orthotopic PDX growth was monitored every week using the Xenogen IVIS Spectrum Imaging System (Caliper Life Sciences, Hopkinton, MA, USA) by intraperitoneal injection of D-luciferin (Sigma-Aldrich, St. Louis, MO, USA), at 150 mg/kg body weight, in saline solution. Imaging was done when tumors reached ~1.0 cm in largest diameter.

### 4.5. Small Animal ImmunoPET/CT Imaging

The ^89^Zr-Df-H3K3 tracer (200 μL, corresponding to 3.7 ± 0.4 MBq or 100 µCi, 0.1–11 nmol) was administered by lateral tail vein injection to restrained NSG mice bearing orthotopic HCC PDX622. Small animal imaging was performed on a Inveon PET/CT system (Preclinical Solutions; Siemens Healthcare Molecular Imaging, Knoxville, TN, USA). This PET/CT system was built in combination, with excellent radial, tangential, and axial resolutions (>1.5 mm). CT was scanned at 80 kVp at 500 μA, 2nd bed position, half scan 220° of rotation, and 120 projections per bed position with a cone beam micro-X-ray source (50 μm focal spot size) and a 4064 × 4064-pixel X-ray detector [32]. Reconstruction of these scanning data was performed using Shepp–Logan filtering and cone-beam filtered back-projection and with the two-dimensional ordered-subset expectation maximization (OSEM 2D) algorithm [33]. PET images were scanned (energy window: 350 to 650 keV) at the various time points after the tracer injection, i.e., 1 and 4 h for 3 min; 24 h for 5 min; 48 and 72 h for 10 min; 96–120 h for 15 min. Tracer uptake by organs was computed from the regions of interest (ROI) and converted in counts per minute (cpm) by the Inveon Research Workplace software (Preclinical Solutions; Siemens Healthcare Molecular Imaging, Knoxville, TN, USA). Finally, the individual organs’ uptake was calculated as percentage-injected dose per gram (%ID/g) from the ROI and the injected dose data.

### 4.6. Biodistribution Study of ^89^Zr-Df-H3K3 

To evaluate the tracer biodistribution in three groups of mice (NSG-ctl: non-tumor, PDX-NSG-blk, and PDX-NSG-nblk; *n* = 4 per group), ^89^Zr-Df-H3K3 (200 μL, corresponding to 3.7 ± 0.4 MBq, 15–16 μg) was administered by tail vein injection at the end of PET imaging (168 h p.i.). Mice were euthanized by CO_2_ gas asphyxiation, and each of the major mouse organs were removed, rinsed in PBS, air dried for 3–5 min, weighed, and radioactivity was counted using a gamma-counter. PDX was dissected from normal liver by visual inspection after harvesting the liver. The dissected liver and PDX tissues were weighed separately and radioactivity was measured using a gamma counter. Tracer uptake by each organ was determined by measuring the total number of cpm. Count data were background subtracted and decay corrected to the time of injection, and the %ID/g for each tissue sample was calculated by normalization to the total activity injected.

### 4.7. Statistical Analysis

Unpaired Student’s *t*-test was used for data comparisons. All *p* values < 0.05 were considered statistically significant. All statistical analyses were performed with PRISM8 software (GraphPad, v8.4.0.2, San Diego, CA, USA).

Detailed methods on Western blotting, immunofluorescence, analytical flow cytometry, immunohistochemistry, in vitro cell binding assay, and immunoreactivity [34] can be found in Supplementary Materials.

## 5. Conclusions

We have developed a humanized anti-GPC3 antibody that demonstrates excellent in vivo capability to identify GPC3-positive HCC tumors, with minimal background from the normal liver or other organs. We believe that ^89^Zr-Df-H3K3 is a highly translatable probe for the specific and high-contrast imaging of GPC3-positive HCCs, which may aid early detection of HCC and allow timely intervention.

## Figures and Tables

**Table 1:** Characterization of Df-H3K3 and ^89^Zr-Df-H3K3 tracer (*n* = 3).

Characterization of Df-H3K3/^89^Zr-Df-H3K3	Results
Purity of Df-H3K3	>98.4 ± 0.5%
Df/H3K3 (c/a) ^a^	1.8 ± 0.2
Radiochemical yield by TLC	70.6 ± 5.1%
Radiopharmaceutical purity by HPLC (mean%±SD)	96.9 ± 2.2
Specific activity (mean ± SD: mCi/mg of Df-H3k3)	5.9 ± 1.6
Immunoreactivity (mean% ± SD)	69.6 ± 3.2

^a^ number of chelators (c) coupled per antibody (a).

## Data Availability

All data generated or analyzed during this study are included in this published article and its supplementary files.

## Supplementary Materials

The following are available online at https://www.mdpi.com/article/10.3390/cancers13163977/s1, Figure S1: Schematic diagram of CDR grafting of mouse 1G12 antibody onto a human IgG scaffold to generate humanized H3K3, Figure S2: Original uncropped Western blot for Figure 1, Figure S3: Comparison of H3K3 and 1G12 binding affinity on GPC3-expressing HepG2 cells, Figure S4: Immunoreactivity of the ^89^Zr-Df-H3K3 tracer, Table S1: Biodistribution of 89Zr-Df-H3K3 into PDX and various mouse organs, Table S2: Comparison of 89Zr-Df-H3K3 tracer uptake in tumor with uptake in liver and muscle.
