# Supplementary material for: A Humanized Anti-GPC3 Antibody for Immuno-Positron Emission Tomography Imaging of Orthotopic Mouse Model of Patient-Derived Hepatocellular Carcinoma Xenografts
Source: Cancers (Basel). 2021 Aug 6;13(16):3977. doi: 10.3390/cancers13163977 (PMC8391944; doi:10.3390/cancers13163977)
Supplement: Supplementary file 1 [file cancers-13-03977-s001.zip › cancers-1294987-supplementary.pdf]

cancers

MDPI

Supplementary Materials: A Humanized Anti-GPC3 Antibody for Immuno-Positron Emission Tomography Imaging of Orthotopic Mouse Model of Patient-Derived Hepatocellular Carcinoma Xenografts

Arutselvan Natarajan, Hui Zhang, Wei Ye, Lakshmi Huttad, Mingdian Tan, Mei-Sze Chua, Sanjiv S. Gambhir and Samuel K. So

Supplemental Methods

Western Blotting, Immunofluorescence, Analytical Flow Cytometry, In Vitro Cell Binding Assay, and Immunohistochemistry (IHC)

Cells were lysed with T-PER Tissue Protein Extraction Reagent (ThermoFisher Scientific, Rockford, IL, USA) supplemented with Halt Proteinase Inhibitor (ThermoFisher Scientific, Rockford, IL, USA) according to the manufacturer's recommendations. Supernatants were collected after spinning down lysates at 12,000 g for 10 min at 4 °C. The protein concentrations of supernatants were measured with Pierce BCA Protein Assay Kit (ThermoFisher Scientific, Rockford, IL, USA). Protein (10 μg) was electrophoresed on NuPAGE 4–12% Bis-Tris gel (Invitrogen, Carlsbad, CA, USA) and transferred to polyvinylidene difluoride (PVDF) membranes and blocked with 10% non-fat milk for 1 h at room temperature and probed with the primary antibody (H3K3, 5 μg/mL), followed by incubating with HRP-anti human Fc (Sigma-Aldrich, St. Louis, MO, USA), 1:1000 dilution with TBS-T (0.05%Tween 20 in TBS buffer) at room temperature for 1 h. The mouse anti-human GPC3 mAb (clone 1G12; BioMosaics Inc., Burlington, VT, USA) and HRP-anti-mouse antibody (Sigma-Aldrich, St. Louis, MO, USA) were used as a positive control antibody for GPC3 detection. GPC3 protein was detected by SuperSignal West Femto Maximum Sensitivity substrate (ThermoFisher Scientific, Rockford, IL, USA). GAPDH, used as an internal loading control, was detected using primary anti-GAPDH antibody (1 μg/mL) (sc-365062, Santa Cruz Biotechnology, Santa Cruz, CA, USA), followed by goat anti-mouse IgG-HRP secondary antibodies (sc-2005, Santa Cruz Biotechnology, Santa Cruz, CA, USA) at 1:1000 dilution.

For immunofluorescence and FACS staining, live cells were incubated with 2 μg/mL H3K3, or non-targeting mouse IgG (Jackson Immuno Research, Inc., West Grove, PA, USA) at 4 °C for 1 h followed by incubation with Alexa 488-conjugated goat anti-human IgG (A1103, Invitrogen, Eugene, OR, USA) at 1:1000 dilution in PBS with 1% goat serum, at 4 °C for 1 h. Fluorescence signal was visualized and recorded with a Nikon Eclipse 80i fluorescence microscope (Nikon Corporation, Tokyo, Japan) and with a Nikon DXM1200f digital camera (Nikon Corporation, Tokyo, Japan). Confocal study was performed with an Inverted Zeiss LSM 880 laser scanning confocal microscope with AiryScan (Zeiss, Germany). FACScan (Becton Dickinson, San Jose, CA, USA) was used for analytic flow cytometry. FACS data was analyzed by FlowJo software.

To compare the binding affinity of the mouse anti-GPC3 antibody, clone 1G12, with its humanized counterpart, clone H3K3, in vitro binding assay was done with human HepG2 cells (HCC cell line with positive GPC3 expression), and human PC3 cells (prostate cells line with negative GPC3 expression [14]. Biotinylated-Antibodies (50 pM–50 nM) were incubated with 1×10^5 cells/200 μL in 1% bovine serum albumin in PBS (PBSA)/well for 90 minutes at 4 °C. Cells were washed thrice and stained with 10 μL/well of Streptavidin-APC (0.2 mg/mL; 17-4317, eBioscience, San Diego, CA, USA), to each of the well containing cells and kept for 1 hour at 4 °C. Finally, stained cells were washed twice and resuspended in PBSA for flow cytometry analyses. Binding experiments were performed in

duplicates. Mean fluorescence intensity (MFI) was computed by subtracting MFI of secondary antibody alone and non-specific antibody (mouse IgG) that did not bind to HepG2 cells, i.e., which does not bind to GPC3. Binding affinity was measured and quantified as equilibrium dissociation constant (KD) using GraphPad software (Prism 8). Computation was performed using non-linear regression curve fitting with one site—total binding equation.

For IHC, tumor tissue sections were fixed with 10% formalin and embedded in paraffin and sectioned at 5 μm thickness. Sections were deparaffinized and rehydrated by washing with 100% xylene, a 1:1 mix of 100% xylene and 100% ethanol, 100% ethanol, 95% ethanol, 70 % ethanol and 50 % ethanol, sequentially, 3 min per wash, followed by rinsing with tap water. Antigen retrieval was done by fully covering the sections with Dako Target Retrieval Solution (S1700, Dako, Carpinteria, CA, USA) and heating at 95 °C for 30 min. Sections were cooled down at room temperature for 30 min and then rinsed three times with PBS to remove the Target Retrieval Solution. Endogenous peroxidase activity was blocked with 3% H_2O_2. H3K3 antibody (20 μg/mL) was added to the sections and incubated at 4 °C overnight followed by incubating with 3.8 μg/mL biotin conjugated goat anti-human IgG (A18809, Invitrogen, Rockford, IL, USA) with 1% goat serum. Staining for 1G12 was performed the same way, using 1G12 antibody (B00R, BioMosaics Inc, Burlington, VT, USA) at 100 times dilution, and goat anti-mouse IgG-HRP (sc-2005, Santa Cruz Biotechnologies, Santa Cruz, CA, USA), diluted 100 times in PBS. Diaminobenzidine (DAB) histochemistry kit (Dako, Carpinteria, CA, USA) with Streptavidin-HRP (554066, BD Pharmingen, San Diego, CA, USA) was used to visualize the immunoreaction with a Nikon Eclipse 80i fluorescence microscope (Nikon Corporation, Tokyo, Japan).

Immunoreactivity of ^{89}Zr-Df-H3K3

Immunoreactivity of ^{89}Zr-Df-H3K3 was determined using the live cell-binding assay as described previously [34], using single cell suspension of GPC3-positive human HCC tumor cells (used to generate PDX622). Each tube containing PDX622 cells (200 μL; ranging from 1–24 million cells per mL in 1% PBSA at pH 7.4) received 100 μL of ^{89}Zr-Df-H3K3 from stock solutions of 5 kBq/18.2 μg/mL. Tubes for the blocking-cell bound experiment received 100-fold excess of cold H3K3 (182 μg in 1 mL) 1 h prior to addition of ^{89}Zr-Df-H3K3. All tubes were vortexed and incubated at 37 °C for 2 h. All tubes for measuring cell bounds activity were centrifuged at 300 g for 3 min, and supernatants were gently removed to measure radioactivity associated with cell pellets, using a gamma counter (1470 WIZARD Automatic Gamma Counter; Perkin Elmer, Waltham, MA, USA). The counts per minute (cpm) data were background corrected for comparison of with the total activity versus bound activity. These data were used to plot linear regression analysis by plotting of total/bound activity on the Y axis versus 1/[normalized cell concentration] on the X axis. Immunoreactivity was calculated as (1/y-intercept) × 100. Non-specific mouse IgG antibody tracer was used as a negative control.

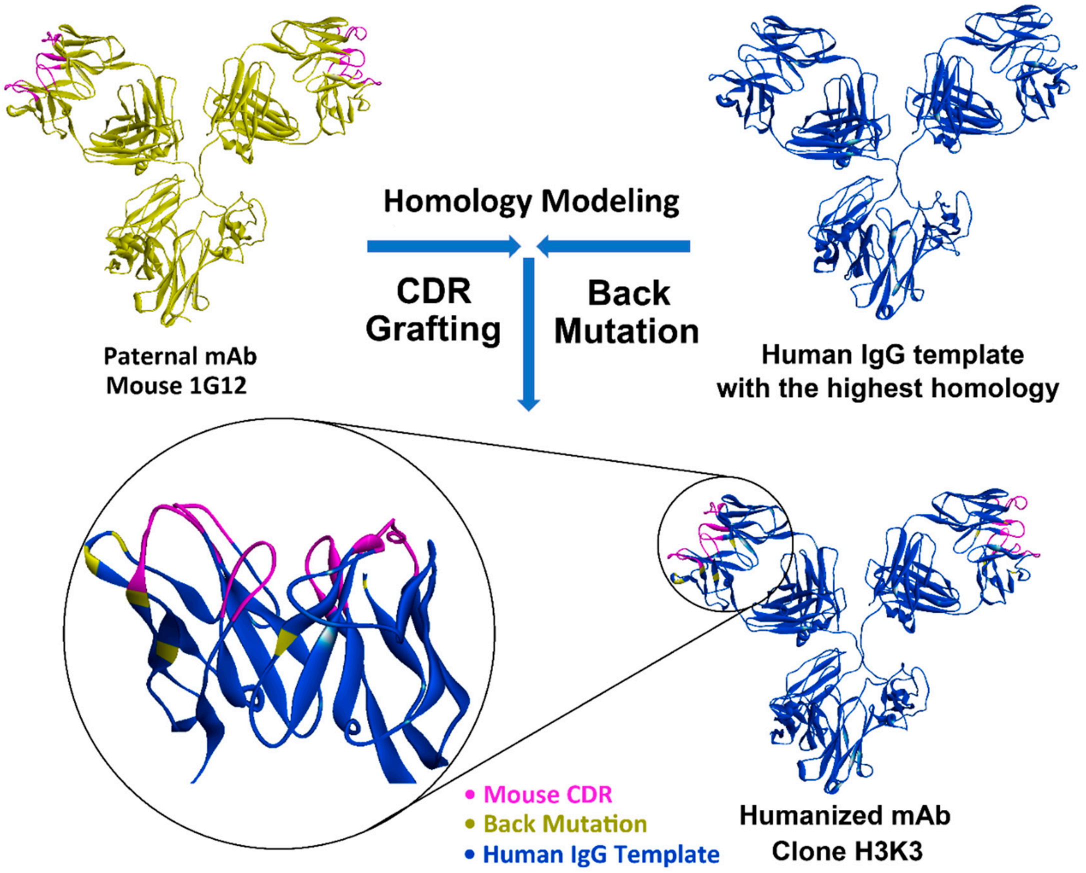

Figure S1. Schematic diagram of CDR grafting of mouse 1G12 antibody onto a human IgG scaffold to generate humanized H3K3. The transplantation of mouse CDRs onto a human framework (FR) usually lead to suboptimal conformations of these binding loops. Therefore, critical mouse framework residues need to be re-introduced as back mutations to restore the optimum CDR orientations for antigen binding. H3K3 was humanized through CDR grafting and proper back mutation, in which the CDR regions and a few framework residues from paternal 1G12 were retained (Yellow: 1G12 sequences; Fuchsia: 1G12 CDRs; Blue: human sequences).

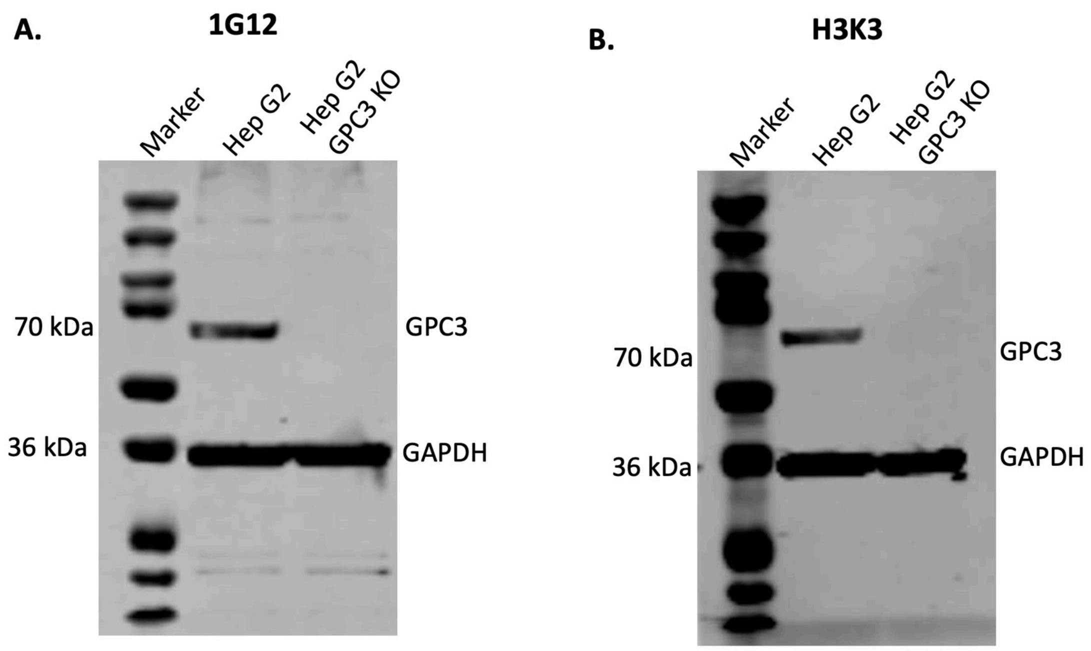

Figure S2. Original uncropped Western blot for Figure 1. Western blot of (**A**) mouse clone 1G12 and (**B**) humanized clone H3K3 staining of GPC3 expression in HepG2 parental cells (GPC3-positive) and GPC3-knockout counterparts (HepG2 GPC3KO; GPC3 negative). GAPDH was used as the internal control.

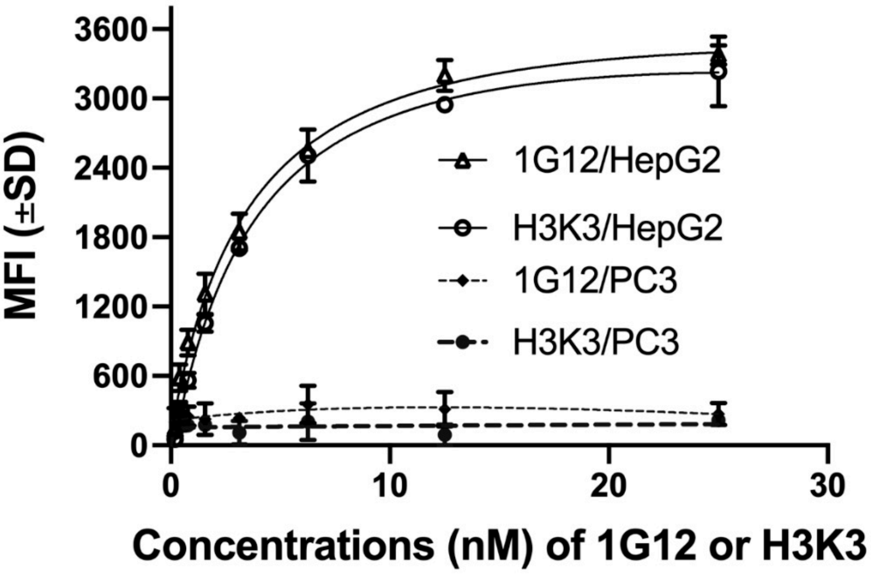

Figure S3. Comparison of H3K3 and 1G12 binding affinity on GPC3-expressing HepG2 cells. Cell binding curves of H3K3 and 1G12 antibodies with GPC3-positive HepG2 cells, and GPC3-negative PC3 cells. Binding experiments were performed in duplicates and repeated twice. Mean fluorescence intensity (MFI) were computed by subtracting MFI signals from secondary antibodies. X axis = concentrations (50 pM to 25 nM) of the antibodies, and Y axis = the MFI (±SD) corresponding to antibody concentrations. Antibodies were incubated with either HepG2 or PC3 cells 2 × 10^5 cells/200 μL in PBSA/well for 90 minutes at 4 °C. Binding affinity was measured and reported as equilibrium dissociation constant (Kd). Kd values are 3.89 ± 0.23 and nM 3.50 ± 0.35 nM (R^2 = 0.99) for H3K3 and 1G12, respectively.

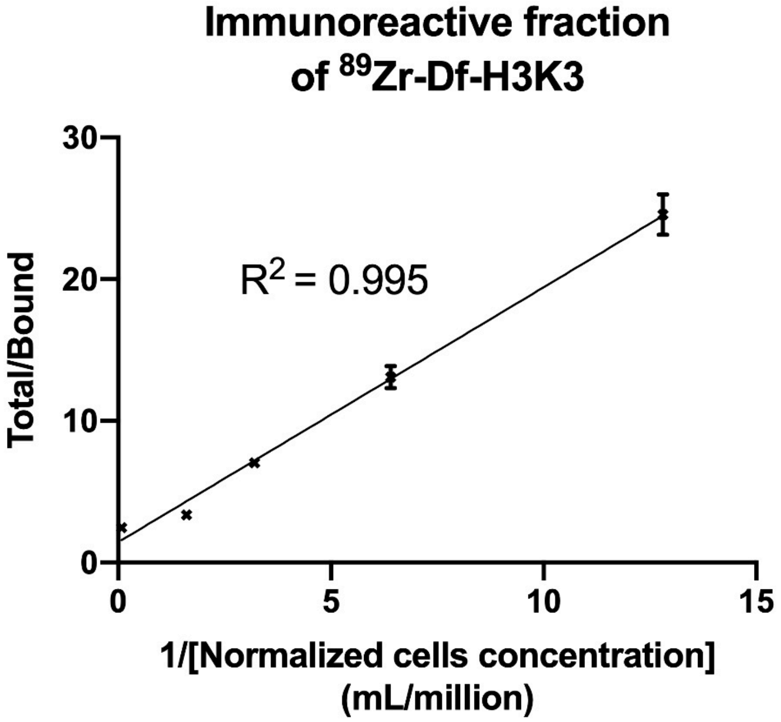

Figure S4. Immunoreactivity of the ^{89}Zr-Df-H3K3 tracer. Immunoreactivity of the ^{89}Zr-Df-H3K3 tracer was performed with GPC3 expressing cells. The assay was set up using multiple concentrations of cells from 12.5 to 0.1 million cells/mL, and the final concentration of tracer was 10 ng/ml. Cells were used at various concentrations to infinite antigen excess (1/y-intercept). A plot of (total/bound) activity against 1/[Normalized cell concentration] in mL/million provided immunoreactive fraction of the tracer. The data presented are corrected with background subtraction of activity from the pre-blocked cells.

Table S1. Biodistribution of ^{89}Zr-Df-H3K3 into PDX and various mouse organs.

Mouse organs	NSG-ctl		PDX-NSG-blk		PDX-NSG-Nblk	
	**Mean (%ID/g)**	**± SD**	**Mean (%ID/g)**	**± SD**	**Mean (%ID/g)**	**± SD**
Blood	0.05	0.02	0.77	0.26	0.09	0.03
Heart	1.02	0.13	1.06	0.28	0.92	0.08
Lungs	1.23	0.44	1.84	0.44	0.86	0.14
Liver	4.94[c]	0.77	7.24	1.05	9.63	1.52
Spleen	9.98	1.38	7.01	1.82	10.61	2.27
Pancreas	0.82	0.11	1.01	0.24	0.85	0.18
Stomach	1.21	0.20	1.19	0.12	0.97	0.23
Intestine	1.97	1.09	2.32	0.59	2.25	0.39
Kidney	2.55	0.45	3.93	0.61	2.80	0.91
Muscle	**0.73**[e]	0.04	0.76	0.08	**0.78**[d]	0.09
Bone	1.91	0.75	2.38	0.19	2.44	0.20
Brain	0.07	0.06	0.09	0.02	0.04	0.01
Tumor	No tumor		5.8[b]	1.02	12.05[a]	1.44

Table S2. Comparison of ^{89}Zr-Df-H3K3 tracer uptake in tumor with uptake in liver and muscle.

Comparison	Formula (refer to Table S1)	Ratio
Nblk tumor *vs* blk tumor	a/b	2.1
Nblk tumor *vs* Ctl-liver	a/c	2.4
Nblk-tumor *vs* muscle	a/d	15.4[f]
Ctl-liver *vs* muscle	c/e	6.8[g]
Nblk-tumor-to-muscle ratio *vs* Ctl-liver-to-muscle ratio	f/g	2.3
